# DEKP: a deep learning model for enzyme kinetic parameter prediction based on pretrained models and graph neural networks

**DOI:** 10.1093/bib/bbaf187

**Published:** 2025-04-24

**Authors:** Yizhen Wang, Li Cheng, Yanyun Zhang, Yujia Cao, Daniyal Alghazzawi

**Affiliations:** School of Computer Science, Hubei University, No. 368 Youyi Road, 430062 Wuhan, China; School of Computer Science, Hubei University, No. 368 Youyi Road, 430062 Wuhan, China; Key Laboratory of Intelligent Sensing System and Security, Hubei University, Ministry of Education, No. 368 Youyi Road, 430062 Wuhan, China; Hubei Key Laboratory of Big Data Intelligent Analysis and Application, Hubei University, No. 368 Youyi Road, 430062 Wuhan, China; School of Computer Science, Hubei University, No. 368 Youyi Road, 430062 Wuhan, China; Key Laboratory of Intelligent Sensing System and Security, Hubei University, Ministry of Education, No. 368 Youyi Road, 430062 Wuhan, China; Hubei Key Laboratory of Big Data Intelligent Analysis and Application, Hubei University, No. 368 Youyi Road, 430062 Wuhan, China; School of Computer Science, Hubei University, No. 368 Youyi Road, 430062 Wuhan, China; Faculty of Computing and Information Technology (FCIT), 3599 King Abdulaziz University (KAU), Unit 3600, Jeddah 22254-7653, Saudi Arabia

**Keywords:** deep learning, enzyme kinetic parameters, graph neural network, pretrained model

## Abstract

The prediction of enzyme kinetic parameters is crucial for screening enzymes with high catalytic efficiency and desired characteristics to catalyze natural or non-natural reactions. Data-driven machine learning models have been explored to reduce experimental cost and speed up the enzyme design process. However, the prediction performance is still subject to significant limitations due to the variance in sequence similarity between training and testing datasets. In this work, we introduce DEKP, an integrated deep learning approach enzyme kinetic parameter prediction. It leverages pretrained models of protein sequences and incorporates enhanced graph neural networks that provide comprehensive representation of protein structural features. This novel approach can effectively alleviate the performance degradation caused by sequence similarity variation. Moreover, it provides sensitive detection of changes in catalytic efficiency due to enzyme mutations. Experiments validate that DEKP outperforms existing models in predicting enzyme kinetic parameters. This work is expected to significantly improve the performance of the enzyme screening process and provide a robust tool for enzyme-directed evolution research.

## Introduction

Improvement of enzyme catalytic efficiency toward specific substrates and identification of appropriate enzymes to catalyze natural or non-natural reactions are key challenges in enzyme engineering. These aspects are essential for downstream tasks such as directed evolution, enzyme screening, understanding reaction mechanisms, and optimizing metabolic pathways design [1, 2]. The turnover number ($ k_{\text{cat}} $) and Michaelis constant ($ K_{\text{m}} $) are the key enzyme kinetic parameters that serve as indicators of enzyme performance in enzyme engineering, reflecting the catalytic capacity and substrate affinity, respectively. Although sequence-based deep learning models have achieved good results in predicting enzyme kinetic parameters, the lack of protein structure data makes it difficult for the models to fully capture the 3D conformation and active site information of enzymes. This limitation restricts the accuracy of predictions and the in-depth understanding of enzyme catalytic mechanisms.

Machine learning methods, which have emerged in genomics [3], drug discovery [4], and protein property prediction [5, 6], have gradually been applied to the prediction of enzyme kinetic parameters. Utilizing machine learning models allows for the rapid and accurate prediction of enzyme-related coefficients without the need for expensive experiments. Some data-driven machine learning models have been applied to cell metabolism analysis in model organisms such as *Escherichia coli* [7, 8] and *Saccharomyces cerevisiae* [9]. These studies typically rely on well-studied but small-scale biological datasets, making it difficult for the models to generalize to other species [10]. Additionally, using complex representations is challenging and further increases the risk of model overfitting. Li *et al*. [11] proposed a deep learning model DLKcat to predict $ k_{\text{cat}} $ based on substrates and protein sequences. Compared to previous models, it overcomes the limitation of only being able to predict $ k_{\text{cat}} $ for model organisms. Nevertheless, its performance declines significantly when enzymes are not similar to those in the training datasets. Kroll *et al*. [12] developed a model named TurNuP, which employs advanced protein pretrained models to represent sequences. The model also introduced reaction fingerprints to simultaneously represent substrate and product, specifically optimizing its prediction performance of wild-type enzyme $ k_{\text{cat}} $ values in cases of low sequence similarity. In earlier work, Kroll *et al*. also utilized UniRep [13] to encode amino acid sequences and combined them with substrate molecular fingerprints to perform predictions of enzyme-substrate binding affinity [14]. In addition to incorporating factors such as pH and temperature [15, 16], Maeda *et al*. [17] developed the $ K_{\text{m}} $ prediction model MLAGO, which only uses simple feature encodings like EC numbers and biological entity IDs to reduce computational costs. By combining feature vectors of proteins and substrates as inputs, these models have achieved better performance compared to DLKcat.

Benefiting from the latest advancements in natural language processing [18, 19], recent studies have increasingly employed large pretrained language models [20, 21] to represent protein sequences. These models are then fine-tuned on various protein property prediction tasks, which has proven to be highly effective. However, the protein 3D structure determines their functional characteristics [22]. The lack of 3D structural information causes these models to rely exclusively on the amino acid sequence, which leads to an inability to accurately identify and locate key functional regions. They also perform poorly when handling enzymes with low sequence similarity. Highly accurate protein structures are now more accessible than ever [23, 24]. Compared to using protein sequences alone, structure information can reveal the geometric arrangement of active sites, which significantly affects catalytic performance [25]. Incorporation of protein structure helps deep learning models better capture the complex relationships between enzymes and substrates, thereby improving the accuracy and reliability of enzyme kinetic parameter predictions.

Here, we present a deep learning model named DEKP to predict enzyme kinetic parameters $ k_{\text{cat}} $ and $ K_{\text{m}} $. DEKP utilizes a graph Transformer [26] improved with graph neural networks (GNNs) [27, 28] to learn representations from protein sequences, structures, and substrates. Additionally, to complement geometric features and extract richer information about protein structure and function, DEKP incorporates secondary structure, dihedral angles, and accessible surface area. We compared four frequently used pretrained models and nine machine learning models, and validated their effectiveness on $ k_{\text{cat}} $ and $ K_{\text{m}} $ datasets classified by enzyme function and enzyme type. Furthermore, we evaluated DEKP’s performance on enzymes with low sequence similarity, which has shown improvements over existing models. We also verified that DEKP can assign higher attention weights to enzyme mutation sites. Finally, we employed DEKP to predict the trends in how mutations at different sites affected enzyme catalytic efficiency, demonstrating its potential to assist in enzyme-directed evolution. Our method is expected to significantly enhance the accuracy and efficiency of enzyme kinetic parameter predictions, which will advance enzyme engineering and high-throughput screening applications.

## Results

### Overview of DEKP

**Figure 1:**
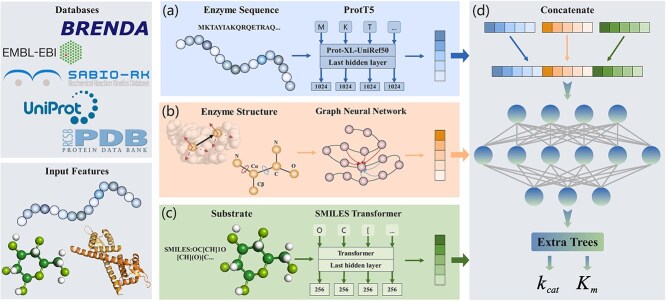
The overview of DEKP. DEKP integrates protein sequences, 3D structures, and substrate SMILES data to predict enzyme kinetic parameters. Protein sequences and substrates are encoded using the pre-trained models ProtT5 and SMILES Transformer, respectively. The protein structure is first encoded as a graph containing spatial and physicochemical information, and then a GNN is used to learn its representation. All representations are concatenated and fed into a fully connected neural network. Finally, DEKP outputs the predicted values of $ k_{\text{cat}} $ and $ K_{\text{m}} $.

DEKP is a deep learning model designed to predict enzyme kinetic parameters ($ k_{\text{cat}} $ and $ K_{\text{m}} $). It integrates information from protein sequences, protein structures, and substrates. The framework of this model is divided into four parts (Fig. 1). Parts (a) and (b) involve representation learning from enzyme sequences and substrate information, respectively. These tasks utilize two pretrained language models, ProtT5-XL-U50 (ProtT5) [29] and SMILES Transformer [30], which represent the amino acid sequences and substrates converted into the simplified molecular-input line-entry system (SMILES) format, respectively. Part (c) focuses on structure representations. In addition to utilizing the 3D structure data, DEKP also incorporates secondary structure, dihedral angles, and accessible surface area. The inclusion of these feature sets provides each residue with explicit secondary structure labels and surface exposure information. Residues or atoms are represented as nodes, and their spatial distances along with biochemical interactions form the edges. This approach effectively transforms the protein into a graph that integrates its structural and physicochemical properties. For protein structure information, DEKP employs an improved Graph Transformer [26]. In part (d), the feature vectors obtained from the three components are concatenated and fed into the extra trees model to predict the values of $ k_{\text{cat}} $ and $ K_{\text{m}} $.

### Prediction performance evaluation for DEKP

**Figure 2:**
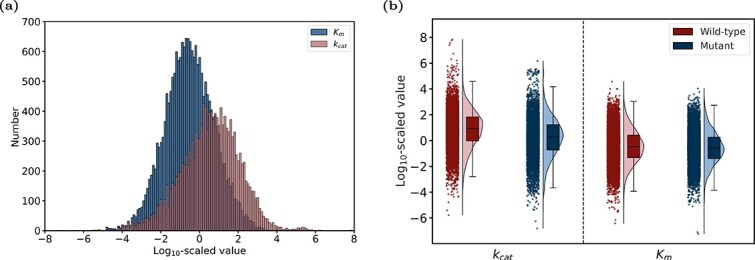
Statistical analysis of datasets. (a) Distribution of $ k_{\text{cat}} $ and $ K_{\text{m}} $ values on a $\log _{10}$ scale. (b) Distribution of enzyme kinetic parameter values for mutant-type and wild-type.

We have constructed two distinct datasets for $ k_{\text{cat}} $ and $ K_{\text{m}} $, containing 13 401 and 19 847 entries, respectively (Fig. 2a). In addition to overall evaluation, the datasets were also categorized into mutant and wild-type enzymes to assess their value distribution (Fig. 2b). The two datasets were randomly split into training, validation, and test sets in proportions of 80%, 10%, and 10%, respectively.

Four frequently used pretrained models were considered. We used ProtT5+MolFormer [31] and ProtT5+SMILES Transformer to represent enzyme sequences and substrates, respectively. The latter combination achieved better results (Supplementary Fig. S2a). We then employed the pst_t33_so model [32] based on esm2_t33_650M_UR50D [33] and the graph ransformer based on GNNs to learn structure representations. To avoid errors from incidental results, we averaged the outcomes after five rounds of testing. Graph Transformer exhibited better prediction results under the condition of incorporating protein sequence data (Supplementary Fig. S2b). Moreover, regardless of which model was used to encode protein structures, the inclusion of structure data consistently led to improved prediction performance. DEKP ultimately used ProtT5 to encode protein sequences, SMILES Transformer to encode substrates, and graph Transformer to encode protein structures. The concatenated feature vectors were then input into the machine learning model.

Previous research has shown that when input feature vectors have high dimensionality, machine learning models may struggle to effectively fit the data, and deep learning models may fail to achieve satisfactory prediction performance due to the need for large amounts of labeled data [16]. Typically, expanding the training dataset can alleviate some of the limitations of deep learning models. However, given the current scarcity of enzyme reaction data, this approach is not feasible in the short term, necessitating the exploration of alternative methods. To address this challenge, we employed a residual multilayer perceptron (MLP) to reduce the feature vectors obtained from pretrained models to a consistent, fixed dimensionality. This dimensionality reduction not only streamlines the feature space but also significantly decreases the computational overhead associated with predicting enzyme kinetic parameters.

**Figure 3:**
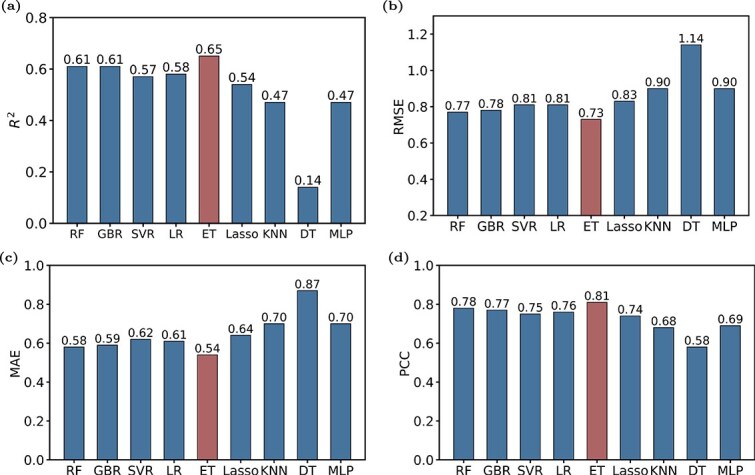
Comparison of prediction performance across different models. Comparison of (a) coefficient of determination ($R^{2}$), (b) root mean square error (RMSE), (c) mean absolute error (MAE), and (d) Pearson correlation coefficient (PCC) of nine diverse machine learning models. The predicted $K_{m}$ values are the average results of randomly splitting the dataset five times. The full names of the machine learning models are as follows: RF: Random Forest; GBR: Gradient Boosting Regressor; SVR: Support Vector Regressor; LR: Linear Regression; ET: Extra Trees; Lasso: Lasso Regression; KNN: K-Nearest Neighbors; DT: Decision Tree; MLP: Multi-Layer Perceptron.

**Figure 4:**
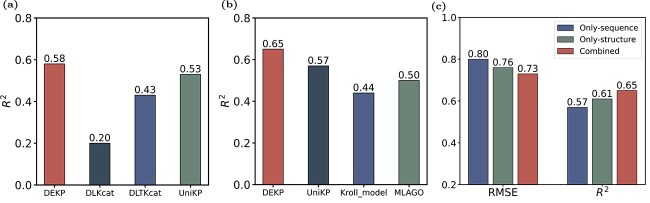
DEKP assessment and comparison with existing models. (a) Comparison of $R^{2}$ values on the $ k_{\text{cat}} $ test dataset for DEKP, DLKcat, DLTKcat, and UniKP. (b) Comparison of $R^{2}$ values on the $ K_{\text{m}} $ test dataset for DEKP, UniKP, Kroll_model, and MLAGO. (c) The $ K_{\text{m}} $ prediction performance when using only sequence, only structure, and sequence and structure as inputs, with the substrate included by default. Protein sequences are encoded using ProtT5, protein structures are encoded using GNN, and substrates are encoded using the SMILES Transformer. All the representation vectors are then fed into extra trees model.

To explore the performance of different machine learning models, we conducted a comprehensive evaluation of nine different machine learning models (Fig. 3). Extra trees achieved the best performance on all the evaluation metrics (ET RMSE = 0.73, $R^{2}$ = 0.64). To assess whether feature dimensionality reduction affected model performance, we also evaluated the non-reduced feature vectors as inputs to the extra trees model, and the results showed minor enhancements compared to the previous performance (ET RMSE = 0.72, $R^{2}$ = 0.65). This model excelled at managing high- and low-dimensional data, demonstrating its ability to fit the data effectively. The extra trees model outperformed other machine learning models in predicting enzyme kinetic parameters due to its superior ability to handle high-dimensional and low-dimensional data, enhanced randomization, and computational efficiency. These strengths make it ideally suited for complex biochemical datasets, ensuring accurate and reliable predictions within the DEKP framework. After evaluating all the models, we assessed the predictive performance using only enzyme sequences, only enzyme structures, and a combination of enzyme sequences and structures (Fig. 4c). The combination of both types of information achieved the best results.

In order to further evaluate the robustness and generalizability of DEKP, we conducted additional experiments focusing on data quality and enzyme source diversity. First, we filtered the protein structure data obtained from the RCSB Protein Data Bank (PDB) [34] by excluding structures with a resolution above 3Å [35]. After retraining the model using this filtered dataset (Supplementary Fig. S8), the *R*^2^ value of DEKP increased to 0.60 and 0.66 on the kcat and Km datasets. It indicated that although the quality of structure data has some impact on prediction accuracy, the model remains highly stable even when processing data with variable quality. Secondly, we analyzed the distribution of enzyme sources in the $ k_{\text{cat}} $ and $ K_{\text{m}} $ datasets, which included 574 and 776 different organisms, respectively. To test whether the model overly depended on frequently studied organisms (such as *Homo sapiens* and *E. coli*), we removed data corresponding to these two organisms. The results revealed that the $R^{2}$ value on the $ K_{\text{m}} $ dataset increased from 0.65 to 0.67 and 0.66 (Supplementary Fig. S7), further demonstrating that the model’s performance does not rely on any specific frequently studied organism.

### Performance comparison with other methods

**Figure 5:**
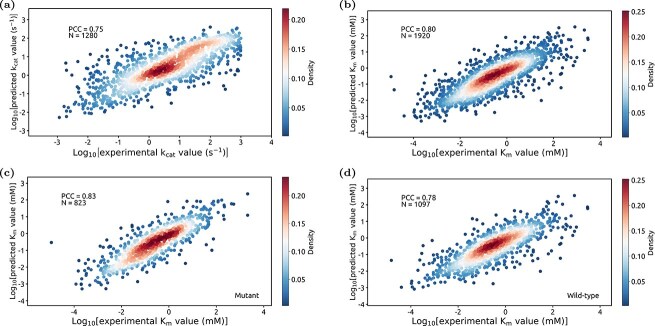
DEKP performance for enzyme kinetic parameter prediction. (a-b) Scatter plots indicate the PCC between experimental and predicted values with density represented by the color gradient on the whole $ k_{\text{cat}} $ and $ K_{\text{m}} $ test datasets. (c-d) The PCC values of DEKP for mutant and wild-type enzymes on the $ K_{\text{m}} $ test dataset.

In order to comprehensively evaluate the accuracy of DEKP in predicting enzyme kinetic parameters, we selected advanced and publicly available models for comparison. We used DLKcat, UniKP-Kcat, and DLTKcat to predict $ k_{\text{cat}} $, and UniKP-Km, Kroll_model along with MLAGO to predict $ K_{\text{m}} $. After introducing protein structure data as one of the inputs, DEKP achieved higher accuracy than the other models when predicting $ k_{\text{cat}} $ ($R^{2}$ = 0.58) (Fig. 4a), attaining a PCC value of 0.75 on the entire test dataset (Fig. 5a). When predicting $ K_{\text{m}} $, the $R^{2}$ value of DEKP was 0.65, remaining higher than those of the other compared models. It achieved a PCC value of 0.80 on the test dataset (Fig. 5b). This indicated that incorporating protein structure data has a positive effect on predicting enzyme kinetic parameters.

It has been demonstrated that the sequence similarity between target enzymes and those in the training set significantly impacts the model’s predictive performance [12]. In other words, lower sequence similarity may lead to poorer predictive results. The lack of spatial structural information may impede the model’s capacity to detect structural variations in enzymes that lead to changes in catalytic efficiency. Consequently, we attempted to integrate 3D structure data to enhance the accuracy and robustness of model prediction. To further evaluate the performance of DEKP at different levels of sequence similarity, we divided the test set into four subsets based on their maximum sequence similarity to the training set: 0%–40%, 40%–80%, 80%–99%, and 99%–100%. After computing values for each subset, we found that DEKP maintained good and stable performance in predicting $ k_{\text{cat}} $ and $ K_{\text{m}} $, exhibiting robustness across varying levels of sequence similarity (Fig. 8a and b). Since MLAGO does not use protein sequences, we did not test it in this experiment. DEKP significantly outperformed DLKcat, DLTKcat, and Kroll_model at all similarity levels. Moreover, at low (0%–40%) and medium-high (80%–99%) levels of sequence similarity, DEKP showed better performance and greater stability than UniKP. Notably, in the low sequence similarity range (0%–40%), the performance of all comparison models dropped substantially, whereas DEKP still outperformed them. This is likely because the model was trained on a larger dataset and incorporated protein structure data as input. The 3D structure of proteins provides the relative spatial positions and interactions of amino acids, which helps identify the active site. This information cannot be directly inferred from the sequence alone. Moreover, the introduction of attention mechanisms enables the model to learn effective features during training. By combining large pretrained language models to learn sequence and substrate representations, DEKP achieved excellent prediction performance.

Nevertheless, it is important to note that the quantity and quality of the dataset largely determine the final training results of the model. We designed the architecture of DEKP for a specific enzyme kinetic parameter prediction task. When the dataset changes, the model must also be adjusted to better suit the new task.

### DEKP performance for enzyme kinetic parameters prediction

**Figure 6:**
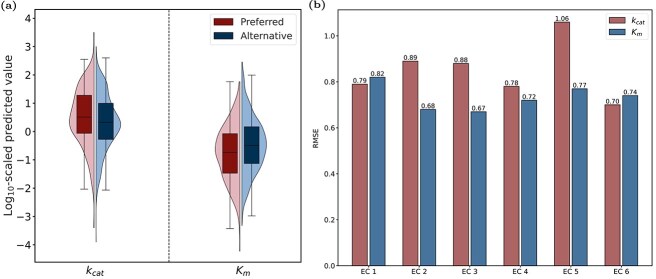
Enzyme promiscuity analysis. (a) Distribution of the $\log _{10}$-scaled $ k_{\text{cat}} $ and $ K_{\text{m}} $ values for preferred and alternative substrates, classified based on experimental $ k_{\text{cat}} $ and $ K_{\text{m}} $ values. (b) The RMSE values of $ k_{\text{cat}} $ and $ K_{\text{m}} $ test datasets classified by EC numbers.

Mutations may significantly affect the catalytic activity of enzymes, and screening enzymes with favorable catalytic performance can help design novel non-natural metabolic pathways for synthesizing industrially important chemicals [36]. We classified the datasets into wild-type and mutant enzymes to assess the discriminatory ability of DEKP. It performed well for both enzyme types (Supplementary Fig. S1). When predicting $ K_{\text{m}} $, it achieved a PCC value of 0.78 for wild-type enzymes (Fig. 5d) and 0.82 for mutant enzymes (Fig. 5c). In addition, we used DEKP to analyze enzyme promiscuity on the test dataset. Enzymes capable of catalyzing multiple substrates were screened [11]. Based on experimental measurements, the substrates with the maximum $ k_{\text{cat}} $ or minimum $ K_{\text{m}} $ were deemed the preferred substrates ($ k_{\text{cat}} $: 256 entries; $ K_{\text{m}} $: 349 entries), while the remaining substrates were regarded as alternative substrates ($ k_{\text{cat}} $: 626 entries; $ K_{\text{m}} $: 757 entries). In Fig. 6a, the predicted $ k_{\text{cat}} $ values from DEKP demonstrated that the preferred substrates (median $ k_{\text{cat}} $ = 0.51) were higher than the alternatives (median $ k_{\text{cat}} $ = 0.32). For the predicted $ K_{\text{m}} $ values, the preferred substrates (median $ K_{\text{m}} $ = –0.74) were lower than the alternatives (median $ K_{\text{m}} $ = –0.49). This indicated that DEKP can detect substrate preferences for promiscuous enzymes.

The first digit of an enzyme’s EC number represents its primary reaction class. To evaluate the prediction performance of DEKP across different enzyme classes, we categorized the enzyme data based on the first digit of the EC number, excluding EC 7 due to insufficient samples (Supplementary Fig. S5). In our dataset, the majority of entries were oxidoreductases (EC 1) and transferases (EC 2). For the $ k_{\text{cat}} $ and $ K_{\text{m}} $ datasets, the EC 6 group and EC 2 group achieved the best performance ($ k_{\text{cat}} $ RMSE = 0.70, $ K_{\text{m}} $ RMSE = 0.68), and the $ K_{\text{m}} $ dataset performed better overall than the $ k_{\text{cat}} $ dataset (Fig. 6b). This experiment demonstrated that the model’s performance does not strictly correlate with the data size. One reason for this is that fitting a small dataset is more susceptible to random factors or outliers, which may create a strong linear relationship in certain cases and lead to model overfitting. Such results may not represent the true trend. Nonetheless, DEKP still demonstrated robust predictive performance on the overall test set.

### Apply DEKP to identify critical mutation sites in enzymes

**Figure 7:**
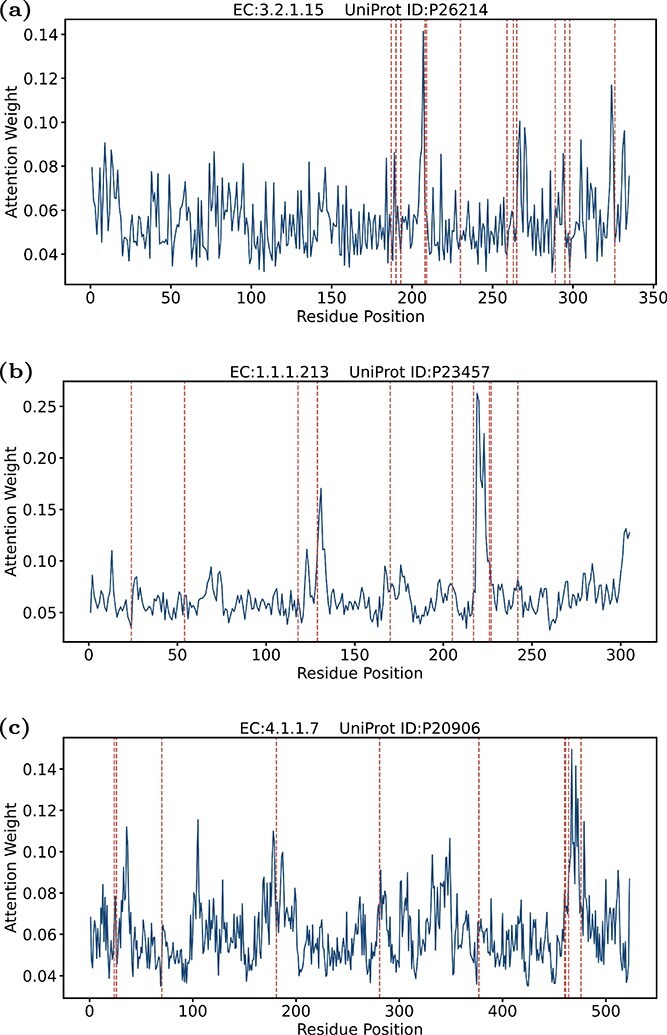
Analysis of the correlation between attention weights and enzyme mutation sites. Attention weight distribution across residue positions of the enzymes (a) P26214; (b) P23457; (c) P20906. Red dashed lines indicate mutation sites.

Enzymes are highly structure-specific proteins, their active sites and mutation sites play critical roles in their catalytic functions. By observing changes in enzyme activity before and after mutations in amino acid residues, the impact on catalytic reactions can be understood. After verifying the prediction accuracy of DEKP for wild-type and mutant enzymes, three enzymes from the training set, each with more than 15 mutations along with their reaction substrates were selected. These enzymes were chosen to assess whether DEKP can focus on mutation sites and assign them higher weights. The UniProt IDs for these three enzymes are P26214, P23457, and P20906. The calculation of attention weights was performed by first averaging the multi-head attention from each layer. The results were then accumulated and normalized at the node level. The observation that mutation sites are tightly distributed or overlap with the peaks of attention weights indicated that the model can give more attention to mutation sites (Fig. 7). However, there are also instances where high attention is observed in regions without annotated mutations. This inconsistency suggests that DEKP may be capturing additional structural or functional features not currently documented in the mutation annotations, or it might reflect noise and incompleteness in the available data. Although our current analysis is qualitative, these observations highlight the complexity of enzyme function and the potential for further investigation into other influential factors. Generally speaking, DEKP can effectively identify mutation sites critical for enzymatic function. Through the accurate identification of these key residue sites, DEKP facilitated a deeper understanding of how mutations alter enzyme activity. Consequently, DEKP has the potential to serve as a reliable tool to predict the effects of mutations on enzyme activity and function, aiding in the design of enzyme engineering strategies and the interpretation of mutational impacts in biochemical pathways.

### Use DEKP to predict the impact of mutations on enzyme catalytic efficiency

**Figure 8:**
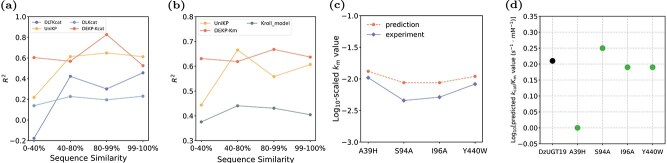
DEKP demonstrates robust performance when predicting mutant enzymes with low sequence similarity and shows potential for enzyme evolution. (a) Comparison of $ R^{2} $ values for DEKP’s prediction of $ k_{\text{cat}} $ against DLTKcat, UniKP, and DLKcat across different levels of sequence similarity. (b) Comparison of $ R^{2} $ values for DEKP’s prediction of $ K_{\text{m}} $ against UniKP and Kroll_model across different levels of sequence similarity. (c) Comparison between experimental values and DEKP’s predicted $ K_{\text{m}} $ values for mutants of the enzyme DzUGT19. (d) DEKP’s prediction of the $ k_{\text{cat}} $/$ K_{\text{m}} $ values for the wild-type enzyme DzUGT19. Black dots represent the experimental values of DzUGT19 as references, while green dots indicate correctly predicted trends in catalytic efficiency of its mutant enzymes.

Enzyme-directed evolution aims to enhance the efficiency and specificity of biosynthetic pathways by optimizing enzyme catalytic performance [37, 38]. Accurately predicting the trends in how different mutation sites affect enzyme catalytic efficiency can accelerate the screening process for high-efficiency enzyme variants. The advent of directed evolution has enabled the development of customized enzymes with superior catalytic activity and stereoselectivity, often surpassing the performance of small molecule catalysts [39]. However, the overall efficiency of the directed evolution process remains constrained by the wet-lab experiments. The glycosyltransferase DzUGT19 can catalyze the conversion of deltonin into zingiberensis newsaponin in the presence of the glycosyl donor uridine diphosphate glucose (UDP-Glc). It has been preliminarily studied to elucidate the biosynthetic pathway of steroidal saponins in Dioscorea zingiberensis [40]. We used DEKP to predict the $ k_{\text{cat}} $ (Supplementary Fig. S3) and $ K_{\text{m}} $ values of the glycosyltransferase to verify whether the model could narrow down the range of candidate variants requiring wet-lab testing and focus on those enzyme variants more likely to exhibit superior performance. The prediction results of DEKP were compared with those of other existing models (Supplementary Tables S1 and S2). Our wet-lab dataset for glycosyltransferases includes five samples that were not present in the training set. Using the wild-type enzyme DzUGT19 as a reference, the $ k_{\text{cat}} $/$ K_{\text{m}} $ values of mutants A39H, I96A, and Y440W decreased, while that of mutant S94A increased. The wet-lab and predicted values of $ K_{\text{m}} $ are shown in Fig. 8c. It can be seen that DEKP accurately predicts the changes in $ K_{\text{m}} $ values caused by different mutation sites. Furthermore, the predicted changes in $ k_{\text{cat}} $/$ K_{\text{m}} $ values relative to the reference sample are completely consistent with the trends observed in the wet-lab measurements (Fig. 8d). This indicated that DEKP can predict the trends in catalytic efficiency of the enzyme resulting from mutations at different sites. Additionally, DEKP has the potential to become a powerful tool for accelerating virtual screening of enzymes and reducing the cost of wet-lab experiments.

## Discussion

The prediction of enzyme kinetic parameters is one of the key tasks in enzyme engineering and can be time-consuming and labor-intensive. To address this issue, we propose a new deep learning model named DEKP. This model uses ProtT5 and SMILES Transformer, two pretrained models, to encode protein sequences and substrates, respectively. Additionally, it employs graph Transformer to encode protein structures. AlphaFold has overcome the challenges of experimentally determining protein structures and limited data, enabling large pretrained language models to accurately capture enzyme–substrate features. After incorporating 3D protein structures, DEKP significantly improved the prediction accuracy of enzyme kinetic parameters by extracting and concatenating various feature vectors, surpassing previously reported models. Moreover, to alleviate computational costs, DEKP reduces the obtained feature vectors to a uniform fixed dimension. This also addresses the issue where some machine learning models struggle to effectively fit high-dimensional features to some extent. Previous studies have demonstrated that protein sequence similarity significantly affects model performance. DEKP exhibits stable performance when handling enzyme data with low sequence similarity and overall outperforms other comparative models.

To optimize prediction performance, a unique design combining pretrained models and an improved GNN model was applied to DEKP. This allows DEKP to achieve improved predictive results for different types of enzymes. For enzymes capable of catalyzing multiple substrates, DEKP was able to identify preferred substrates with higher $ k_{\text{cat}} $ values or lower $ K_{\text{m}} $ values. DEKP has the ability to reveal critical enzyme mutation sites impacting catalytic efficiency. When faced with enzymes containing more than 15 mutations, DEKP could assign higher attention weights to the mutation sites. This advantage enabled DEKP to accurately predict the trends in how mutations at different sites of the glycosyltransferase DzUGT19 affected enzyme catalytic efficiency, which demonstrated its potential to assist enzyme-directed evolution. Previous studies typically used descriptors or fingerprints designed by human experts such as RDKit [41] to represent chemical molecules. In contrast, pretrained models have the ability to capture richer and more subtle molecular features. When representing protein structures, each amino acid residue is directly used as a node in a graph, and the edges between nodes are defined based on spatial distances and geometric relationships. This approach is naturally suited to capturing 3D contacts and interactions between protein residues. Compared to relying solely on 1D protein sequences or precomputed contact maps, this method is more adaptable to structural variations. Additionally, the graph Transformer model introduces a self-attention mechanism from Transformers compared to traditional GNNs. This allows the model to easily capture more fine-grained graph topological features when updating each node’s representation, thereby balancing the flexible expression of local and global features. Moreover, by augmenting the existing dataset with experimentally measured inhibitory constants ($ K_{\text{i}} $) and relevant experimental condition annotations, our model could readily be adapted to predict enzyme–inhibitor interactions and $ K_{\text{i}} $. Therefore, DEKP shows promising applications in predicting small molecule inhibitory properties and in the development of novel potential drugs.

DEKP has achieved excellent performance in predicting enzyme kinetic parameters, but there is still room for improvement. Previous studies have shown that AlphaFold2 is typically challenged in predicting 3D structures of proteins with a length longer than 1000. Although these proteins account for only 1.3% of our dataset, the model may experience performance degradation when predicting long proteins. Additionally, considering that most enzyme reactions in existing databases do not specify pH and temperature, we decided not to include these factors in order to maintain the size of the training dataset. Although these factors may potentially affect the prediction accuracy. Furthermore, we did not account for the processing of oligomeric proteins. Our study primarily targets industrial enzyme design, where the influence of oligomerization is relatively minor compared to pharmaceutical applications. Nonetheless, incorporating oligomerization considerations may further enhance the model’s robustness. Developing artificial intelligence-assisted high-throughput methods for predicting enzyme kinetic parameters is a significant challenge in synthetic biology. This model aims to provide an accurate and efficient prediction method to reduce the wet-lab costs associated with enzyme design and directed evolution. Although DEKP has achieved better performance than previous models, there is an urgent need for models that are even more precise and widely applicable to various downstream tasks. Incorporating more wet-lab data and integrating the latest algorithms are promising research directions for the future.

## Methods

### Data preparation

We initially obtained enzyme reaction data by utilizing the application programming interface (API) provided by the BRENDA [42] and SABIO-RK [43] databases, which store extensive enzyme information and biochemical reaction kinetics data. The acquisition and cleaning of $ k_{\text{cat}} $ and $ K_{\text{m}} $ followed similar rules, only complete entries with valid values were retained ($ k_{\text{cat}}>0 $ or $ K_{m}>0 $ and SMILES strings without ”.”). Enzyme types were categorized as either ”wild-type” or ”mutant”. Each record was uniquely identified by the Enzyme Commission (EC) number, substrate, enzyme type, and organism. After removing duplicate records, only the maximum $ K_{\text{m}} $ or $ k_{\text{cat}} $ value was kept for each record. The SMILES strings and compound identifiers (CID) corresponding to each substrate were obtained from the PubChem database [44], which stores a vast amount of compound information. For entries with available UniProt IDs, protein sequences were retrieved using the API provided by the UniProt database [45]. For entries lacking UniProt IDs, protein sequences were obtained using the BRENDA API based on EC numbers and organism information. For enzymes labeled as mutants, the protein sequences were modified based on mutation site information to reflect the amino acid changes. Associated PDB IDs were then extracted based on UniProt IDs. FASTA sequences corresponding to the specified PDB IDs were downloaded from the RCSB PDB, which stores a large number of experimentally determined biomolecular structures. The sequence information of each chain was parsed from these FASTA files. Global sequence alignment was performed to calculate the alignment scores and percentage identities of all associated PDB sequences. The PDB ID with the highest similarity exceeding 90% to the target UniProt sequence was selected, and the corresponding protein structure was retrieved from the database. If no corresponding protein structure was found, it was obtained from the AlphaFold database [46]. After collecting all protein structure data, PDBFixer [47] was used to repair proteins lacking complete structural information. All data underwent multiple rounds of cleaning and were ultimately integrated into a high-quality dataset containing EC numbers, organisms, SMILES strings, substrates, CIDs, protein sequences, UniProt IDs, enzyme types, values, units, and protein structures.

The final dataset comprised 13 401 unique $ k_{\text{cat}} $ entries and 19 847 unique $ K_{\text{m}} $ entries. The $ k_{\text{cat}} $ dataset covered 1930 enzyme structure data entries, of which 954 were from the RCSB PDB and 976 from the AlphaFold database. The $ K_{\text{m}} $ dataset encompassed 3906 enzyme structure data entries, with 1425 from the RCSB PDB and 2481 from the AlphaFold database. All $ k_{\text{cat}} $ and $ K_{\text{m}} $ values were converted to logarithmic scales. The cd-hit-2d [48] tool was used to calculate the similarity between the test set and the training set. The dataset was randomly partitioned multiple times into training, validation, and test sets in proportions of 80%, 10%, and 10%, respectively.

### Protein representation

#### Protein sequence encoding

Within the MPEK framework, ProtT5 is employed for encoding protein sequences. ProtT5 is a protein language model based on the Transformer architecture, which extracts significant features from protein sequences through self-supervised learning. ProtT5 comprises an encoder and a decoder, totaling up to 3 billion parameters. The encoder transforms protein sequences into context-aware embedding vectors, while the decoder is utilized to predict and reconstruct the masked regions within the sequences. Each amino acid is represented as a 1024-dimensional feature vector in the final hidden layer, followed by average pooling. The ultimate protein representation is a 1024-dimensional vector.

#### Protein structure encoding

Inspired by the current state-of-the-art deep learning models for protein design in processing protein structure data [49, 50], we incorporate and encode tertiary and secondary structure data. The encoding of protein structures into graph representations follows the subsequent procedure: (i) parsing protein PDB files and extracting atomic coordinates, (ii) constructing adjacency relationships, (iii) extracting geometric features of proteins, and (iv) building and storing graph data structures. Key atomic coordinates for each amino acid residue, including $N,C_{\alpha }$, $C$, $O$ and $C_{\beta }$ based on the other backbone atoms are extracted. To construct adjacency relationships, spatial relationships between residues are described by calculating the Euclidean distances between key atoms and encoding these distances using radial basis functions (RBF) to achieve smooth and locality-sensitive distance representations: 


(1) \begin{align*}& RBF(d) = \exp \left(-\left(\frac{d-\mu}{\sigma}\right)^{2} \right)\end{align*}

where $ d_{ij} = \| r_{i} - r_{j} \| $ represents the Euclidean distance between atoms, $ \mu $ is the center value, and $ \sigma $ is the width. An adjacency graph between residues is then constructed based on the coordinates of $C_\alpha $. By setting a distance radius $r$ and a maximum number of neighbors $n_{neighbor}$, each residue $i$ is connected by an edge to residue $j$ in the graph if the distance between them is less than or equal to 10Å and within the neighbor limit [51]. Each residue is connected to a maximum of 20 neighboring nodes, even if more nodes exist within radius $r$, only the closest ones are selected. For node features, the extraction of geometric features includes secondary structure types, residue dihedral angles, accessible surface area, and the orientation of residue side chains. Secondary structure types are converted into vectors using one-hot encoding, dihedral angles are encoded as sine and cosine vectors, and the spatial orientation of residue side chains is determined by calculating the direction vectors of side-chain atoms relative to $C_\alpha $ and normalizing them: 


(2) \begin{align*}& d_{i} = \frac{X_{i,\text{atoms}} - X_{i,C_\alpha}}{\| X_{i,\text{atoms}} - X_{i,C_\alpha} \|}\end{align*}

where $ X_{i,\text{atoms}} $ denotes the coordinates of side-chain atom $ i $. For the edges, in order to capture the spatial direction and relative rotational relationship between residues, we construct a local coordinate system for each residue by computing a direction matrix $ O_{i} $: 


(3) \begin{align*} u_{i} &= \frac{X_{i,C_\alpha} -X_{i,N}}{\| X_{i,C_\alpha} - X_{i,N} \|}, v_{i} = \frac{X_{i,C_\alpha} -X_{i,C}}{\| X_{i,C_\alpha} - X_{i,C} \|}, \nonumber\\ b_{i}& = \frac{u_{i} - v_{i}}{\| u_{i} - v_{i} \|}, n_{i} = \frac{u_{i} \times v_{i}}{\| u_{i} \times v_{i} \|} \end{align*}

(4) \begin{align*} & O_{i} = [b_{i}, n_{i}, b_{i} \times n_{i}]\qquad\qquad\qquad\qquad\qquad \end{align*}

where $X_{i,C_\alpha}\!, X_{i,C}, X_{i,N} \in \mathbb{R}^{3}$ represents the coordinates of the $C_\alpha, C, N$ atoms of the $ i $th residue. Then we represent the edge features using sequence-index positional encoding, local distance, direction, and orientation: 


(5) \begin{align*}& e_{ij} = \left( PE(i - j), RBF\left(\| X_{j} - X_{i} \| \right)\!, O_{i}^{T} \frac{X_{j} - X_{i}}{\| X_{j} - X_{i} \|}, q(O_{i}^{T} O_{j}) \right)\end{align*}

For the edge features, the first component applies the Transformer’s sinusoidal positional encoding to the difference in sequence indices of residues $ i $ and $ j $. The second component encodes the distance between nodes using RBF. The third component projects the normalized direction vector onto the local coordinate system of the nodes to obtain the relative direction between the nodes. The final component completes the orientation encoding by converting the spatial rotation matrix into a quaternion.

### Substrate representation

The SMILES Transformer is utilized for substrate encoding. This model encodes the SMILES sequences of chemical molecules to learn molecular-level representations. Initially, the SMILES sequence is segmented into symbols and subjected to one-hot encoding. These vectors are then combined with positional encoding and used as input to the Transformer. The model comprises four Transformer blocks, each containing four multi-head attention mechanisms, an embedding dimension of 256, and two fully connected layers. After the self-attention processing by the Transformer encoder, the model generates contextual vectors for each symbol, capturing rich information within the molecular sequence. To obtain an overall molecular representation, the model performs mean pooling and max pooling on these symbol-level representations and integrates the outputs from the first symbol of the final layer and the penultimate layer. These results are concatenated, ultimately producing a 1024-dimensional molecular fingerprint representation.

### Model architecture

The core components of DEKP comprise pretrained models and GNN to learn representations from proteins and substrates. For a given protein structure graph, modeling is performed using a graph Transformer enhanced by GNN. Within the multi-head attention mechanism, the input node features $h_{i} \in \mathbb{R}^{F}$ undergo linear transformations to generate Query, Key, and Value vectors. For each attention head $ l \in \{1, 2, \ldots , L\} $, the attention scores between nodes are computed and normalized as follows: 


(6) \begin{align*}& \alpha_{i,j}^{l} = softmax\left( \frac{W_{Q}^{(l)} h_{i}^{(l-1)} \left( W_{K}^{(l)} h_{j}^{(l-1)} + W_{E}^{(l)} e_{i,j} \right)}{\sqrt{d}} \right)\end{align*}

where $ W_{Q}^{(l)}, W_{K}^{(l)}, W_{V}^{(l)} \in R^{F \times d} $ denote the independently learnable weight matrices for the $ l $th attention head, and $ W_{E}^{(l)} \in R^{F \times d} $ is the weight matrix utilized for integrating edge features. $ F $ represents the node feature dimension, and $ d $ denotes the feature dimension of each attention head. The normalized attention weights are then used to perform a weighted sum of the value vectors of neighboring nodes, yielding the aggregated features for each head. The outputs from all heads are then concatenated and passed through a linear transformation to obtain the final node representations: 


(7) \begin{align*} & h_{i}^{l} = \sum_{j \in N(i)} \alpha_{i,j}^{l} \left(W_{V}^{(l)} h_{j}^{l-1} + W_{E}^{(l)} e_{i,j}\right) \end{align*}

(8) \begin{align*} & h_{i} = Concat \left(h_{i}^{1}, h_{i}^{2}, \ldots, h_{i}^{L}\right) W_{O}\ \ \end{align*}

where $W_{O} \in \mathbb{R}^{F \times (L \cdot d)}$. To alleviate the vanishing gradient problem, the node features from the previous layer $h_{i}^{(l-1)}$ are added to the features obtained from the current layer through the multi-head attention mechanism $h_{i}^{(l)}$. The updated features are further subjected to a position-wise feed-forward network for additional nonlinear transformations. A global pooling operation is then performed to incorporate the global information of the entire graph into each node’s features. Dimensionality reduction of the feature vectors extracted by the pretrained model is achieved using a residual MLP. The fully connected layers are trained end-to-end via gradient descent using a supervised loss to learn effective feature fusion and dimensionality reduction. Hyperparameters were optimized during training (Supplementary Table S3). Finally, the extra trees model is selected to capture the relationships between the concatenated vectors of enzyme sequences, structures, substrate SMILES, and enzyme kinetic parameters. For building and training models, Python 3.10.14 with PyTorch 2.1.2 and CUDA 11.8 were implemented. DEKP was trained by a NVIDIA RTX A5000 with 24G memory.

### Evaluation metrics

To objectively and effectively evaluate the performance of our model, we employed multiple evaluation metrics. These metrics include the coefficient of determination ($R^{2}$, Eq. 9), Pearson correlation coefficient (PCC, Eq. 10), root mean squared error (RMSE, Eq. 11), and mean absolute error (MAE, Eq. 12). 


(9) \begin{align*} & R^{2} = 1 - \frac{\sum_{i=1}^{n} \left( y_{ie} - y_{ip} \right)^{2}}{\sum_{i=1}^{n} \left( y_{ie} - \bar{y}_{e} \right)^{2}} \qquad\qquad\qquad\ \ \ \end{align*}

(10) \begin{align*} & PCC = \frac{1}{n} \frac{\sum_{i=1}^{n} \left( y_{ie} - \bar{y}_{e} \right) \left( y_{ip} - \bar{y}_{p} \right)}{\sqrt{\sum_{i=1}^{n} \left( y_{ie} - \bar{y}_{e} \right)^{2}} \sqrt{\sum_{i=1}^{n} \left( y_{ip} - \bar{y}_{p} \right)^{2}}} \end{align*}

(11) \begin{align*} & RMSE = \sqrt{\frac{\sum_{i=1}^{n} \left( y_{ie} - y_{ip} \right)^{2}}{n}} \qquad\qquad\qquad\qquad\quad\end{align*}

(12) \begin{align*} & MAE = \frac{\sum_{i=1}^{n} \left| y_{ie} - y_{ip} \right|}{n} \ \qquad\qquad\qquad\qquad\qquad \end{align*}

where $y_{ie}$ denotes the experimentally measured values, $y_{ip}$ represents the model-predicted values, $\bar{y}_{e}$ is the mean of the experimental measurements, and $\bar{y}_{p}$ is the mean of the model predictions.

**Key Points**

- We utilized pretrained models and improved graph neural networks to represent proteins and substrates, which enhanced the accuracy of enzyme kinetic parameter prediction.
- DEKP demonstrated robust performance on enzymes with low sequence similarity.
- It can be further used to identify mutation sites and improve the virtual screening process.

## Supplementary Material

DEKP_supplementary_file_bbaf187

## Data Availability

The source code and data are openly available at https://github.com/wang-yi-zhen/DEKP.
