# Supplementary material for: DEKP: a deep learning model for enzyme kinetic parameter prediction based on pretrained models and graph neural networks
Source: Brief Bioinform. 2025 Apr 24;26(2):bbaf187. doi: 10.1093/bib/bbaf187 (PMC12021017; doi:10.1093/bib/bbaf187)
Supplement: DEKP_supplementary_file_bbaf187 [file dekp_supplementary_file_bbaf187.docx]

# DEKP: a deep learning model for enzyme kinetic parameter prediction based on pretrained models and graph neural networks

Yizhen Wang^1^, Li Cheng^1,2,3,*^, Yanyun Zhang^1,2,3,*^, Yujia Cao^1^, Daniyal Alghazzawi^4^

^1^School of Computer Science, Hubei University, No. 368 Youyi Road, 430062, Wuhan, China

^2^Key Laboratory of Intelligent Sensing System and Security (Hubei University), Ministry of Education, No. 368 Youyi Road, 430062, Wuhan, China

^3^Hubei Key Laboratory of Big Data Intelligent Analysis and Application, Hubei University, No. 368 Youyi Road, 430062, Wuhan, China

^4^Faculty of Computing and Information Technology (FCIT), King AbdulAziz University (KAU), 21589, Jeddah, Saudi Arabia


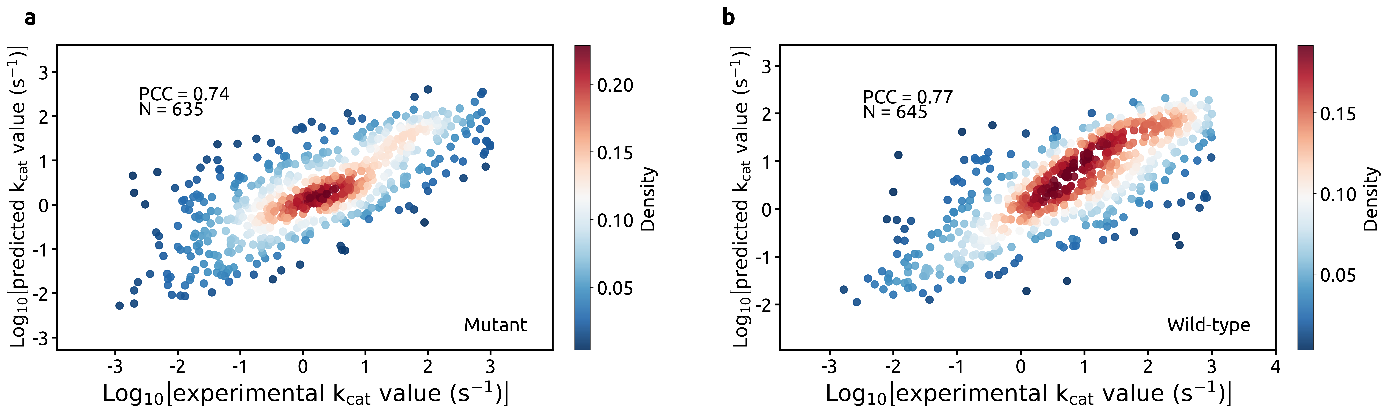


Figure S1. DEKP performance for enzyme kinetic parameter prediction. (a) Pearson correlation coefficient (PCC) of DEKP on the $k_{cat}$ test dataset for mutant enzymes. (b) PCC of DEKP on the $k_{cat}$ test dataset for wild-type enzymes.


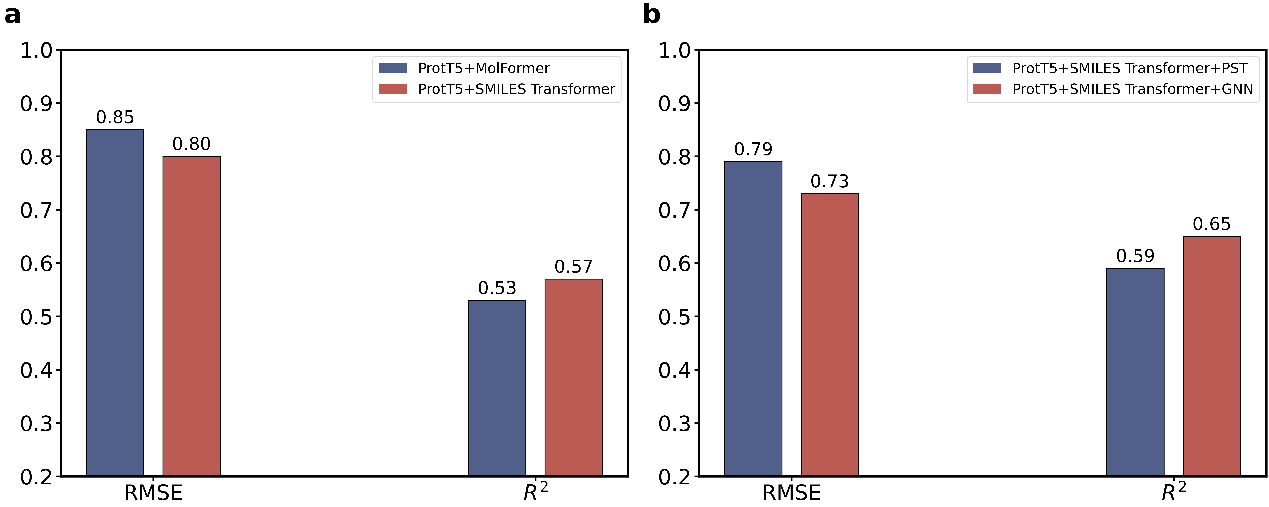


Figure S2. Performance comparison of different pretrained model combinations on test datasets. The predicted $K_{m}$ values are the average results of randomly splitting the dataset five times.


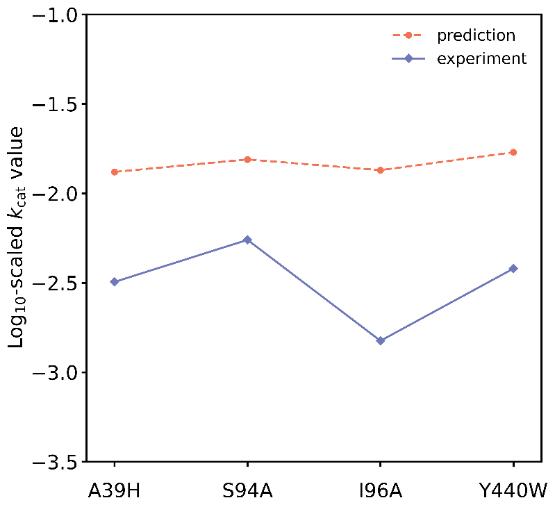


Figure S3. Comparison between experimental values and DEKP’s predicted $k_{cat}$ values for mutants of the enzyme DzUGT19.


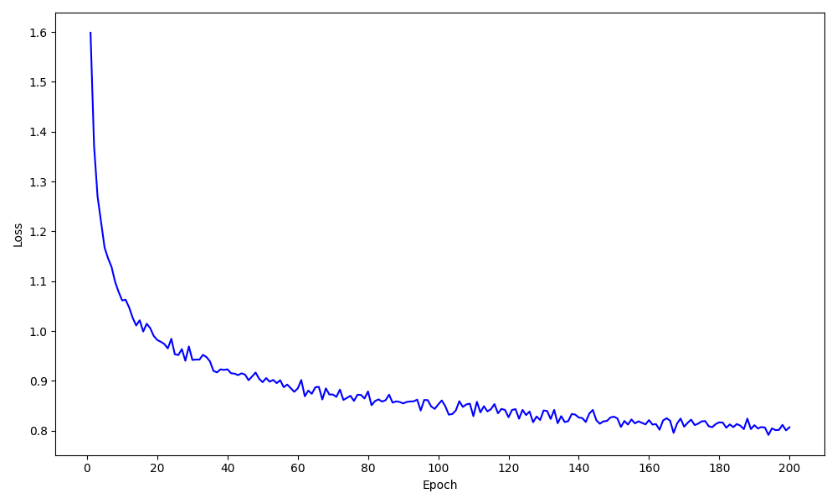


Figure S4. The loss of $K_{m}$ prediction during the training process. The loss of at the end of the training is around 0.78. The loss value is calculated using the mean squared error (MSE) loss function.


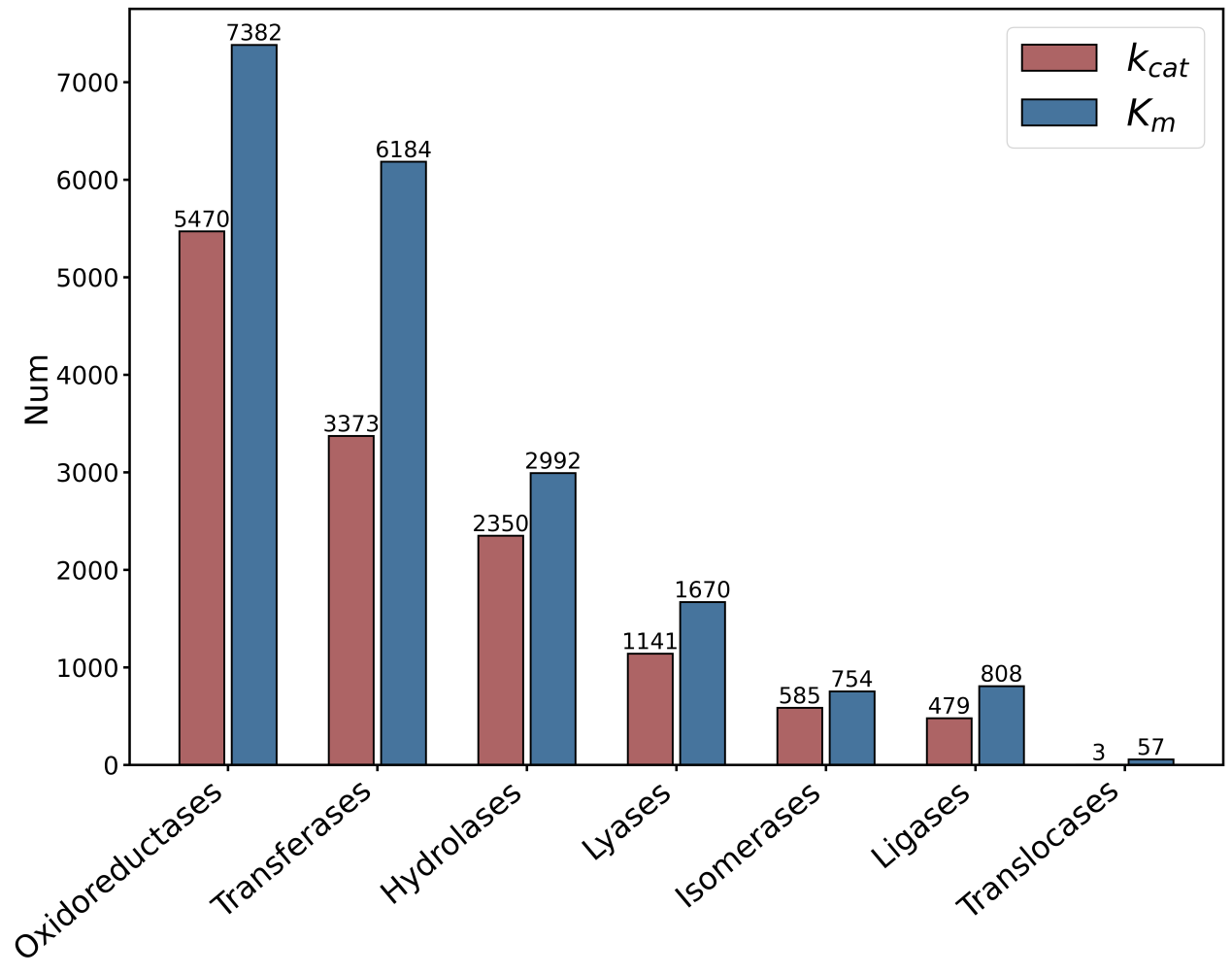


Figure S5. The number of each enzyme class in $k_{cat}$ and $K_{m}$ datasets. Enzyme classes are categorized based on the first digit of their EC number.


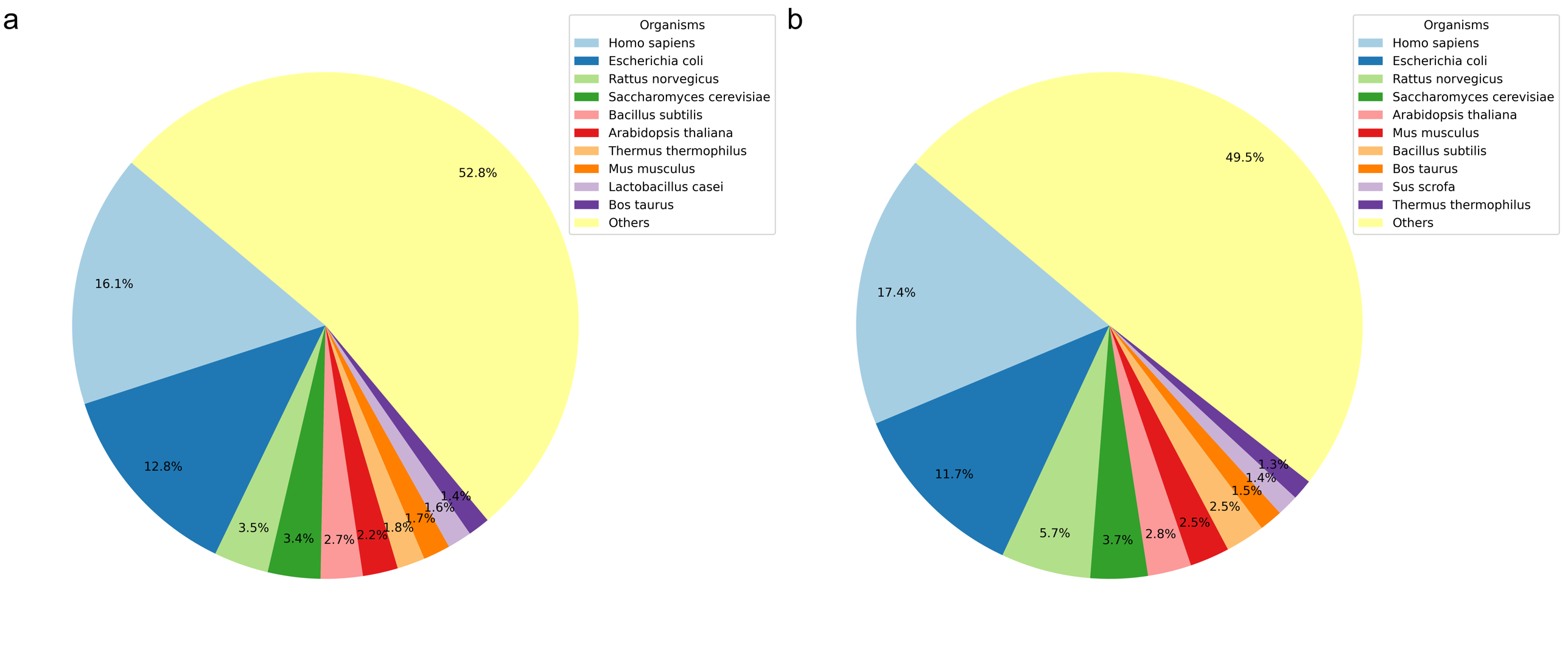


Figure S6. The distribution of enzyme source organisms in the $k_{cat}$ (a) and $K_{m}$ (b) datasets. Top ten organism types are listed with the highest proportions, all other types account for less than 1%.


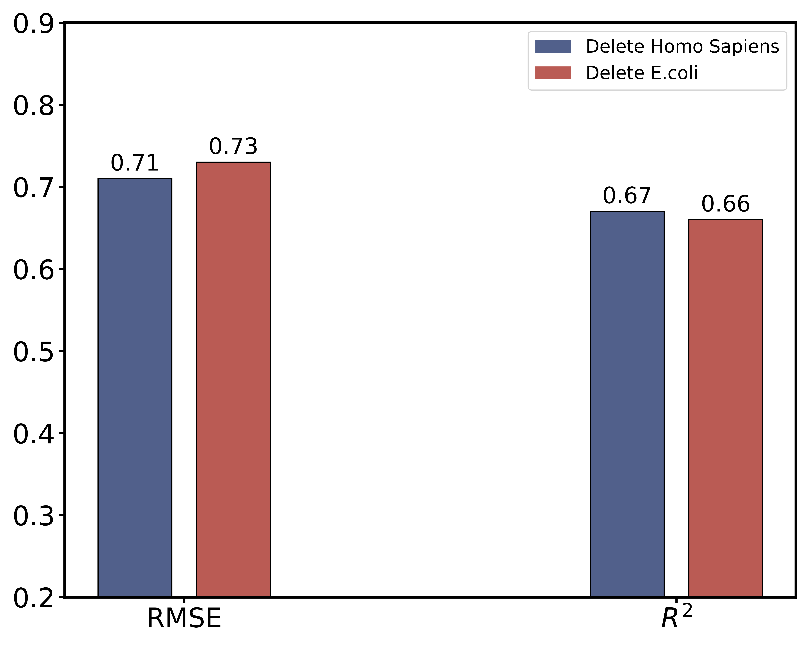


Figure S7. Performance comparison of DEKP after separately removing the organism types Homo sapiens and Escherichia coli from the $K_{m}$ dataset.


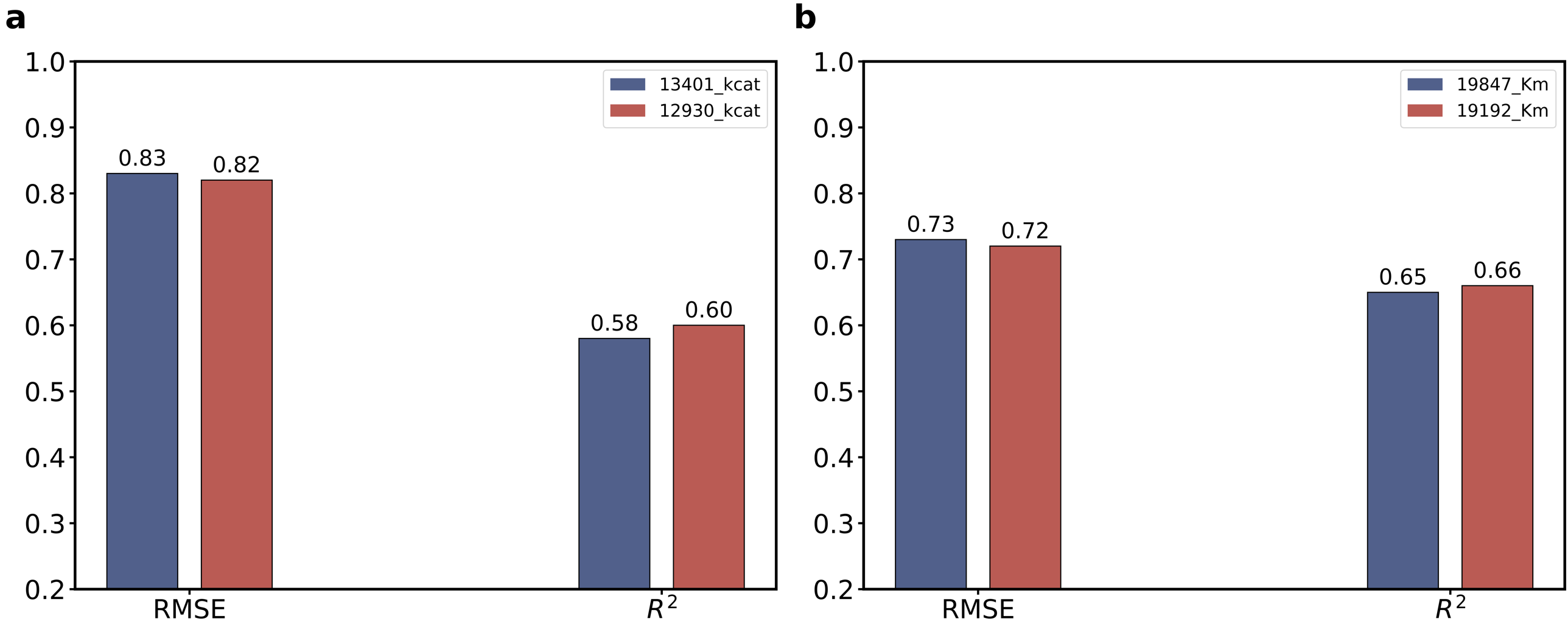


Figure S8. Performance comparison of DEKP after removing protein structure data with a resolution above 3Å from the $k_{cat}$ and $K_{m}$ datasets, respectively. Among these, 13401_kcat and 19847_Km represent the original datasets, while 12930_kcat and 19192_Km represent the datasets after removal.

Table S1. Comparison between experimental values and DEKP’s predicted $K_{m}$values for mutants of the enzyme DzUGT19.

| $k_{cat}$ | Experimental $k_{cat}$ | DLKcat | DLTKcat | TurNup | UniKP | DEKP |
| --- | --- | --- | --- | --- | --- | --- |
| DzUGT19(A39H) | -2.49 | 0.47 | 0.13 | -0.66 | -0.02 | -1.88 |
| DzUGT19(S94A) | -2.26 | 0.50 | 0.05 | -0.70 | -0.01 | -1.81 |
| DzUGT19(I96A) | -2.82 | 0.50 | 0.16 | -0.66 | -0.02 | -1.87 |
| DzUGT19(Y440W) | -2.42 | 0.54 | 0.05 | -0.82 | 0.01 | -1.77 |

Table S2. The experimental determination of $K_{m}$ values for the DzUGT19 enzyme mutants, and the comparison of the $K_{m}$ value prediction results from the DEKP model and other existing models.

| $K_{m}$ | Experimental $K_{m}$ | Kroll_model | UniKP | DEKP |
| --- | --- | --- | --- | --- |
| DzUGT19(A39H) | -1.98 | -1.43 | -1.42 | -1.88 |
| DzUGT19(S94A) | -2.34 | -1.45 | -1.42 | -2.06 |
| DzUGT19(I96A) | -2.29 | -1.47 | -1.41 | -2.06 |
| DzUGT19(Y440W) | -2.08 | -1.44 | -1.41 | -1.96 |

Table S3. The process of DEKP hyperparameters tuning.

| Hyperparameters | Optimization process |
| --- | --- |
| Learning rate | 1e-5, 5e-5, 1e-4, 5e-4, 1e-3, 5e-3 |
| Batch size | 8, 16, 32, 64, 128, 256 |
| Epoch | 10, 30, 50, 90, 100, 150, 200 |
| Hidden size | 16, 32, 64 |
| Kernel | 3, 5, 7, 9 |
| Dropout rate | 0, 0.1, 0.2, 0.3, 0.4, 0.5 |
| Layer | 1, 2, 3, 4, 5 |

Reference

[1] H. Yu, H. Deng, J. He, J. D. Keasling, and X. Luo, “UniKP: a unified framework for the prediction of enzyme kinetic parameters,” *Nat. Commun.*, vol. 14, no. 1, p. 8211, Dec. 2023, doi: 10.1038/s41467-023-44113-1.

[2] A. Kroll, Y. Rousset, X.-P. Hu, N. A. Liebrand, and M. J. Lercher, “Turnover number predictions for kinetically uncharacterized enzymes using machine and deep learning,” *Nat. Commun.*, vol. 14, no. 1, p. 4139, Jul. 2023, doi: 10.1038/s41467-023-39840-4.

[3] F. Li *et al.*, “Deep learning-based kcat prediction enables improved enzyme-constrained model reconstruction,” *Nat. Catal.*, vol. 5, no. 8, pp. 662–672, Jun. 2022, doi: 10.1038/s41929-022-00798-z.

[4] S. Qiu, S. Zhao, and A. Yang, “DLTKcat: deep learning-based prediction of temperature-dependent enzyme turnover rates,” *Brief. Bioinform.*, vol. 25, no. 1, p. bbad506, Nov. 2023, doi: 10.1093/bib/bbad506.

[5] E. C. Alley, G. Khimulya, S. Biswas, M. AlQuraishi, and G. M. Church, “Unified rational protein engineering with sequence-based deep representation learning,” *Nat Methods*, vol. 16, no. 12, pp. 1315–1322, Dec. 2019, doi: 10.1038/s41592-019-0598-1.
